# Collaboration between DELLA proteins and the Mediator subunit MED15 to regulate transcription in plants

**DOI:** 10.1093/jxb/erae393

**Published:** 2024-09-12

**Authors:** Poonam Panchal, Rashmi Ranjan Sutar, Rekha Agrawal, Jitendra K Thakur

**Affiliations:** International Centre for Genetic Engineering and Biotechnology, New Delhi 110067, India; International Centre for Genetic Engineering and Biotechnology, New Delhi 110067, India; International Centre for Genetic Engineering and Biotechnology, New Delhi 110067, India; International Centre for Genetic Engineering and Biotechnology, New Delhi 110067, India; University of Warwick, UK

**Keywords:** Arabidopsis, co-activators, DELLA, Mediator, transcription factors

**Gibberellins (GAs) are diterpenoids that are categorized as one of the main hormones that promote major developmental responses such as germination and stem elongation in plants. DELLA proteins act as the key repressors of GA responses. They interact with hundreds of different proteins. While the functioning of DELLAs as transcriptional co-activators has also been reported earlier, the actual mechanism still remains elusive. A recent report describes interaction of DELLA with the Mediator subunit MED15 as one of the mechanisms contributing to its transcription activation capability (** **Hernández-García *et al.*, 2024** **). Interestingly, this DELLA–MED15 module-mediated transcription regulation seems to be a very ancient conserved mechanism from the bryophyte *Marchantia polymorpha* to the dicot *Arabidopsis thaliana***.

Production of semi-dwarf rice and wheat varieties with reduced lodging and higher grain yields contributed significantly to the ‘Green Revolution’. Interestingly, many genes that show association with semi-dwarf phenotypes encode DELLA proteins. DELLA (aspartate–glutamate-leucine–leucine–alanine residues) proteins are a subgroup of the GRAS [named after GIBBERELLIC-ACID INSENSITIVE (GAI), REPRESSOR of GAI (RGA), and SCARECROW (SCR)] family of transcriptional regulators (Hirsch and Oldroyd, 2009). Usually, they act as negative regulators of GA signaling. The most intrinsically studied aspect of GA functioning in plants is its ability to affect plant phenotype by ‘inhibiting the inhibitors’ (Shani *et al.*, 2024). GA is sensed through a nucleus-localized GID1 (GA INSENSITIVE DWARF1) receptor. When GA binds to the C-terminal domain of GID1, its N-terminal domain forms a lid-like structure over the C-terminal pocket. This whole structure forms a hydrophobic surface for optimum binding of DELLA, which also resides in the nucleus. The GRAS domain of DELLA then interacts with the F-box protein SLEEPY1/GID2 which subsequently leads to proteasomal degradation of DELLA protein (Hernández-García *et al.*, 2021; Shani *et al.*, 2024).

Usually, DELLA proteins function in three different ways: (i) repression of transcription by obstructing interaction of transcriptional activators with their target promoters (Li *et al.*, 2019); (ii) activation of transcription by engaging transcription factors (TFs) with promoters (Yoshida *et al.*, 2014); and (iii) activation of transcription by expropriating transcription repressors away from the promoters (Van De Velde *et al.*, 2017). Despite knowing so much about the functioning of DELLA proteins, there is hardly any information on how they collaborate with other cofactors. Also, the mechanism by which DELLA proteins recruit the transcription machinery on the target promoters has not been explored yet.

**Fig. 1.**
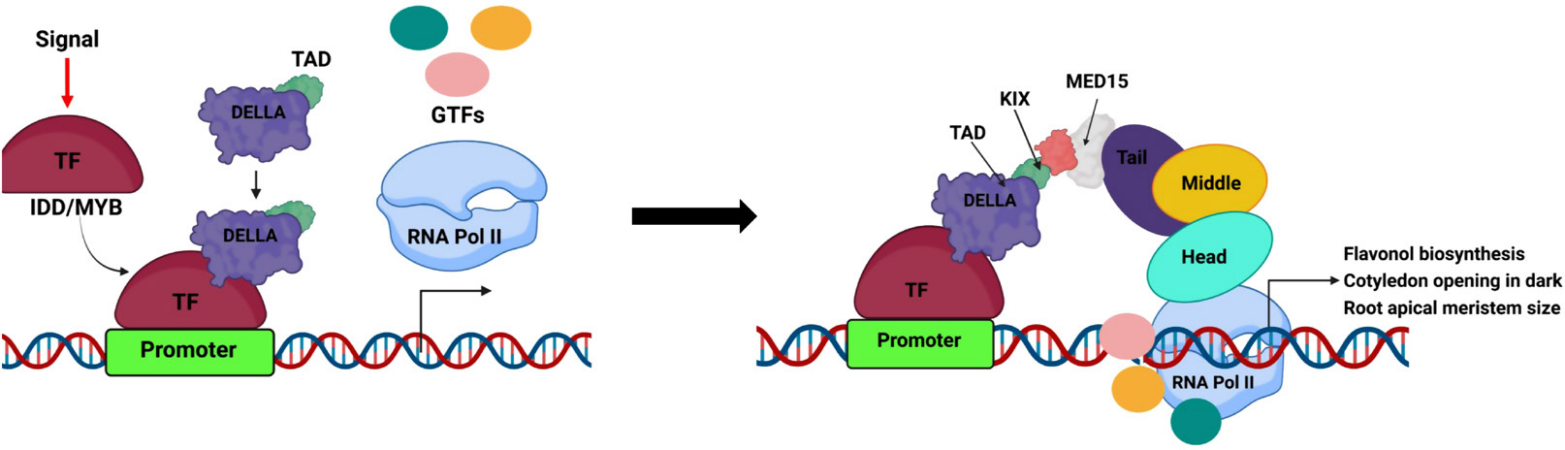
Model explaining the interaction of DELLA with MED15 for transcriptional co-activation. Transcription factors (such as IDD, MYB, etc.) bound to the promoter elements recruit DELLA at the promoter. Then, DELLA and MED15 interact through their TAD and KIX domains, respectively, for the recruitment of the Mediator complex as MED15 is a part of the Mediator complex. The Mediator complex then facilitates recruitment of the transcription machinery (RNA Pol II and GTFs) at these promoters. Thus, docking of the 9AA TAD of DELLA into the KIX domain of MED15 functions as an adapter to connect the TF with RNA Pol II. In the absence of MED15, DELLA cannot recruit the transcriptional machinery at these promoters and transcription is impeded. Interaction of the DELLA 9AA TAD with the MED15 KIX has been shown to activate transcription of genes involved in flavonol biosynthesis, cotyledon opening in the dark, and maintenance of the root apical meristem. The figure is modified from Hernández-García *et al.* (2024). MYB, Myeloblastosis; IDD, indeterminate domain; GTF, general transcription factors. Created by Biorender.com.

In 2014, Yoshida *et al.* reported interaction of DELLA with IDD (INDETERMINATE DOMAIN) TFs to regulate the expression of the *SCL3* (*SCARECROW-LIKE 3*) gene (Yoshida *et al.*, 2014). Thus, DELLA functions as a co-activator for IDD to enhance periclinal cell division in root endodermis. Similarly, DELLA acts as a co-activator for Subgroup 7 Myb (Myeloblastosis viral oncogene homolog) TFs to regulate flavonol biosynthesis, contributing to GA-regulated root growth phenotype (Tan *et al.*, 2019). Hernández-García *et al.* (2019) delineated the functional domains of DELLA proteins and found that the co-activator activity resides towards the N-terminus. In the αD helix of the N-terminal domain, they discovered a nine amino acid transcriptional activation domain (9AA TAD) that is found in a number of TFs including zinc-cluster TFs in fungi and NF-κB, VP16, and p53 in animals (Piskacek *et al.*, 2016). Just like these 9AA TAD-containing TFs, DELLA proteins also function as a hub and interact with hundreds of proteins. In yeast and animals, 9AA TADs are known to interact with the kinase-inducible domain-interacting domain- (KIX) carrying proteins (Thakur *et al.*, 2014; Piskacek *et al.*, 2016). In plants, KIX domains are found in p300/CBP family histone acetyl transferases (HATs), recQ protein-like 5 helicase (RecQL5), and in the Mediator subunit MED15 (Thakur *et al.*, 2013). Interestingly, like DELLA proteins, MED15 also interacts with many different proteins; at least 47 proteins in rice and 57 proteins in Arabidopsis (Dwivedi *et al.*, 2019; Maji *et al.*, 2024). Many of these interactions discovered in rice and Arabidopsis involve 9AA TADs of TFs and the KIX domain of MED15. However, the physiological relevance of 9AA TAD–KIX interaction in plants has not been studied so far. In that regard, a recent report by Hernández-García *et al.* (2024) for the first time, explained the molecular and physiological significance of the interaction between the DELLA 9AA TAD and the MED15 KIX domain (Fig. 1). They found that a subset of DELLA-dependent genes require MED15 for their transcription. Promoters of these genes harbor responsive elements for DELLA-interacting TFs such as IDDs and MYBs. So, the DELLA–MED15 module becomes critical for transcriptional responses of these TFs. For example, flavonol biosynthesis, which is regulated by MYB12, is affected by the DELLA–MED15 module. Phenotypes such as defective cotyledon opening under dark conditions and impaired reduction of the RAM (root apical meristem) are also dependent on DELLA–MED15 interaction (Fig. 1). This interaction is important for the occupancy of Mediator on the promoters of DELLA-regulated genes such as *SCL3* and GA biosynthetic genes. Altogether, in Arabidopsis, DELLA–MED15 interaction is required by a set of TFs to recruit Mediator and the transcriptional machinery on the target promoters to activate transcription.

Since the Mediator complex is modular in nature, interaction of DELLA proteins with other Mediator subunits (such as MED5 and MED8) might lead to the regulation of another set of DELLA-requiring TFs. Dwivedi *et al.* (2019) found that MED15a positively regulates the seed size in rice through the KIX domain, and that a GRAS TF interacts with the KIX domain of MED15a. So, in-depth investigations of coordination between GA signaling, GRAS domain-containing DELLA proteins, Mediator subunits including MED15, and other signaling pathways will provide a more comprehensive idea of regulatory networks that could help in crop improvement.

Although interaction of 9AA TAD of TFs with the KIX domain of MED15 is conserved across eukaryotic kingdoms, the functional relevance of this interaction was explained only in yeasts (Thakur *et al.*, 2008, 2009) and animals (Houser *et al.*, 2023), but not in plants. Observations described in recent studies (Dwivedi *et al.,* 2019; Hernández-García *et al.*, 2024) suggest that 9AA TAD–KIX interaction is also biologically relevant in angiosperms. Hernández-García *et al.* (2024) study in the liverwort *Marchantia polymorpha* showed that the mechanism of transcription co-activation by DELLA via MED15 has been adapted by plants during evolution. Since bryophytes are considered as the first group of plants to evolve among land plants, the Mediator–RNA Pol II recruitment by the DELLA–MED15 module via the 9AA TAD–KIX domain interaction seems to be a conserved mechanism of transcription regulation of genes in land plants.

In plants, participation of DELLA proteins is not confined only to GA signaling. Since DELLA proteins interact with several proteins, they play an important role in crosstalk between different signaling pathways (Hou *et al.*, 2013; Li *et al.*, 2019). For instance, DELLA interacts with EIN3/EIL1 (ETHYLENE INSENSITIVE 3/EIN3-LIKE 1) to establish coordination between GA and ethylene signaling to control apical hook formation in Arabidopsis (An *et al.*, 2012). Similarly, DELLA proteins establish crosstalk between abscisic acid (ABA) and GA by interacting with the TFs ABI3 (ABA-INSENSITIVE3) and ABI5 to control seed germination during stress conditions (Lim *et al.*, 2014). In rapeseed, tomato, and rice, interaction of DELLA with ABA signaling TFs has also been demonstrated for GA and ABA crosstalk regulating growth and development of plants under stress conditions (Shohat *et al.*, 2020; Wu *et al.*, 2020; Liao *et al*., 2023). In response to pathogen attack, DELLA proteins foster JA (jasmonic acid) signaling by interacting with JAZ (jasmonic acid ZIM domain) repressors (Hou *et al.*, 2010). It seems that DELLA proteins promote resistance towards necrotrophic pathogens by modulating JA signaling (Navarro *et al.*, 2008). In contrast to JA signaling, DELLA proteins are negative regulators of SA (salicylic acid) signaling in Arabidopsis (Navarro *et al.*, 2008). However, the relationship between SA and JA with respect to DELLA is not always the same. While in Arabidopsis, these two hormones show antagonism, in rice they show synergism in their interaction with DELLA (De Vleesschauwer *et al.*, 2016). The MYC TFs are known to interact with MED25 and MED17 to regulate JA signaling, whereas MED15 is involved in SA response (Canet *et al.*, 2012; Çevik *et al.*, 2012; Agrawal *et al.*, 2022). Other Mediator subunits such as MED8 and MED16 are also important for JA signaling as they interact with TFs such as FAMA and WRKY33, respectively, to regulate plant defense (Wang *et al.*, 2015; Li *et al.*, 2018). Therefore, it would be of interest to consider the DELLA–Mediator interplay in the context of signaling crosstalk especially during stress responses. It would be useful to discern TFs which might regulate gene expression in response to hormonal signals through the DELLA–Mediator module. Altogether, the discovery of the DELLA–Mediator interaction has provided more avenues for the understanding of transcription regulation in response to environmental cues in plants.
